# Body Fat Distribution, Glucose Metabolism, and Diabetes Status Among Older Adults: The Multiethnic Cohort Adiposity Phenotype Study

**DOI:** 10.2188/jea.JE20200538

**Published:** 2022-07-05

**Authors:** Gertraud Maskarinec, Phyllis Raquinio, Bruce S. Kristal, Adrian A. Franke, Steven D. Buchthal, Thomas M. Ernst, Kristine R. Monroe, John A. Shepherd, Yurii B. Shvetsov, Loïc Le Marchand, Unhee Lim

**Affiliations:** 1 University of Hawaii Cancer Center, Honolulu, HI, USA; 2 Brigham and Women’s Hospital and Harvard Medical School, Boston, MA, USA; 3 University of Maryland, Baltimore, MD, USA; 4 University of Southern California, Los Angeles, CA, USA

**Keywords:** type 2 diabetes, body fat distribution, visceral fat, ethnicity

## Abstract

**Background:**

As the proportion of visceral (VAT) to subcutaneous adipose tissue (SAT) may contribute to type 2 diabetes (T2D) development, we examined this relation in a cross-sectional design within the Multiethnic Cohort that includes Japanese Americans known to have high VAT. The aim was to understand how ectopic fat accumulation differs by glycemic status across ethnic groups with disparate rates of obesity, T2D, and propensity to accumulate VAT.

**Methods:**

In 2013–2016, 1,746 participants aged 69.2 (standard deviation, 2.7) years from five ethnic groups completed questionnaires, blood collections, and whole-body dual X-ray absorptiometry and abdominal magnetic resonance imaging scans. Participants with self-reported T2D and/or medication were classified as T2D, those with fasting glucose >125 and 100–125 mg/dL as undiagnosed cases (UT2D) and prediabetes (PT2D), respectively. Using linear regression, we estimated adjusted means of adiposity measures by T2D status.

**Results:**

Overall, 315 (18%) participants were classified as T2D, 158 (9%) as UT2D, 518 (30%) as PT2D, and 755 (43%) as normoglycemic (NG), with significant ethnic differences (*P* < 0.0001). In fully adjusted models, VAT, VAT/SAT, and percent liver fat increased significantly from NG, PT2D, UT2D, to T2D (*P* < 0.001). Across ethnic groups, the VAT/SAT ratio was lowest for NG participants and highest for T2D cases. Positive trends were observed in all groups except African Americans, with highest VAT/SAT in Japanese Americans.

**Conclusion:**

These findings indicate that VAT plays an important role in T2D etiology, in particular among Japanese Americans with high levels of ectopic adipose tissue, which drives the development of T2D to a greater degree than in other ethnic groups.

## INTRODUCTION

Excess body weight predicts the development of type 2 diabetes (T2D),^1^ but body fat distribution (ie, the presence of visceral adipose tissue [VAT] vs subcutaneous adipose tissue [SAT]) and non-alcoholic fatty liver disease (NAFLD) also appear to play major roles.^2^ This mechanism appears to be particularly important among individuals with Japanese and other Asian ancestry,^3^ as shown in a systematic review of cross-sectional studies that reported a 2 to 4-fold higher risk to develop T2D associated with VAT estimated by Dual X-Ray Absorptiometry (DXA).^4^ Due to its location mainly in the mesentery and omentum, VAT drains directly to the liver through the portal vein and contains a larger number of inflammatory and immune cells and a greater percentage of large adipocytes.^5^ As a result, VAT adipocytes are more metabolically active and more insulin-resistant than adipocytes in SAT. The ratio of VAT to SAT, a metric of body fat distribution, is correlated with cardiometabolic risk and describes the propensity to store fat viscerally in contrast to subcutaneous fat stores.^6^ A “lipid overflow ectopic fat model” proposes that surplus energy is primarily stored as SAT but that energy can be deposited as VAT when the subcutaneous depot is dysfunctional or insufficient.^7^^,^^8^

Not only have higher rates of T2D in individuals of Asian, Pacific Islander, Latino, and African ancestry than whites been documented in many populations,^9^^–^^11^ the association of obesity with T2D also appears to be stronger among Asians than in whites.^12^ At the same time, levels of VAT and NAFLD differ across ethnic groups with lower values in African Americans and higher values among Asians and Native Hawaiians.^13^ The mean VAT was 45% and 73% greater in Japanese American men and women when compared with African Americans among elderly adults in the Multiethnic Cohort (MEC).^13^ Based on the hypothesis that the presence of VAT is associated with hyperglycemia and T2D, this cross-sectional analysis examines how VAT, SAT, their ratio, and percent liver fat, are associated with T2D. The objective of the current analysis was to understand how ectopic fat accumulation differs across the continuum of glycemic status across different ethnic groups with disparate propensity to accumulate VAT and liver fat. Therefore, we compared five ethnic groups including a large number of Japanese Americans within the Adiposity Phenotype Study (APS), a subset of the MEC.

## METHODS

### Study population

In 2013–2016, APS participants from five ethnic groups with similar proportions of normal weight, overweight, and obese were recruited from the MEC, an on-going prospective study in Hawaii and Los Angeles, California that examines diet, lifestyle, and genetic risk factors for cancer.^13^ The MEC consists of more than 215,000 men and women from five ethnic groups (Japanese American, white, Latino, African American, and Native Hawaiian), aged 45–75 years who completed a 26-page questionnaire by mail in 1993–1996.^14^

Selection for the APS study prioritized individuals who reported only one ancestry (except for Native Hawaiians).^13^ Strict eligibility criteria to avoid altered body fat distribution patterns due to disease or medication were applied: body mass index (BMI) outside 18.5–40 kg/m^2^; smoking in the past 2 years due to possible metabolic changes affecting the adipose tissue; soft or metal body implants or amputation; insulin or thyroid medications; and serious medical conditions.^13^ Approximately equal numbers of participants were recruited by sex, race/ethnicity and six BMI strata (18.5–21.9, 22–24.9, 25–26.9, 27–29.9, 30–34.9, and 35–40 kg/m^2^) to optimize the adjustment for total adiposity in populations with different body sizes. The participation rate among eligible cohort members contacted was 23% due to the extensive time requirements for participation.^13^ The protocols were approved by the Institutional Review Boards at University of Hawaii (UH; CHS# 17200) and University of Southern California (USC; #HS-12-00623); all participants provided informed consent.

### Data collection

Eligible cohort members visited study clinics to take part in anthropometric measurements, Magnetic Resonance Imaging (MRI) and Dual X-Ray Absorptiometry (DXA) scans, overnight fasting blood sample collection, and extensive questionnaires assessing nutritional and lifestyle risk factors as well as comorbidities and selected medications. The quantitative food frequency questionnaire with over 180 food items included ethnic-specific foods and relied on a food composition table specific to the MEC.^15^^,^^16^ Total energy intake, alcohol consumption, and diet quality according to the Healthy Eating Index (HEI)-2010, which reflects the 2010 Dietary Guidelines for Americans with higher scores indicating better adherence, were computed from the diet history.^17^ Questionnaire information about average time spent in sleep and different activities on a typical day was used to estimate hours of moderate and vigorous activity per day in hours.

A whole-body DXA scan (Hologic Discovery A at UH and USC) was used to measure total and regional body composition.^13^^,^^18^ To assess localized VAT and SAT areas at the four cross sectional lumbar sites (L1-L2, L2-L3, L3-L4, L4-L5), abdominal MRI scans were obtained on 3-Tesla scanners (Siemens TIM Trio, Erlangen, Germany; software version VB13 at UH; General Electric HDx, Milwaukee, WI, USA, software release 15M4 at USC) using axial gradient-echo sequence with water-suppression and breath-hold (25 slices, 10 mm thickness, 2.5 mm gap, TR/TE = 140/2.6 ms, 70° flip angle). Percent liver fat was estimated from a series of axial triple gradient-echo Dixon-type scans (10 mm slices, no gap, TE = 2.4, 3.7 and 5.0 ms, TR = 160 ms, 25° flip angle) by measuring and analyzing in-phase, out-of-phase, and in-phase signals in a manually selected circular region selected for not including hepatic veins or biliary ducts.^18^

Using the overnight fasting blood samples, insulin was assessed by an ELISA kit (EMD Millipore, Burlington, MA, USA) and glucose by a Cobas Mira Plus Chemistry autoanalyzer (Randox Laboratories, Crumlin, United Kingdom). The Homeostatic Model Assessment of Insulin Resistance (HOMA-IR) was calculated as (fasting insulin (mU/L) × fasting glucose (mg/dL))/405.

### Statistical analysis

Of the 1,861 APS participants, the following exclusions were made: no DXA image (*N* = 21), no MRI image (*N* = 60), no glucose (*N* = 7), unknown fasting hours (*N* = 28) or <8 hours of fasting (*N* = 3). As a result, this analysis included 1,746 participants. In addition to BMI, DXA-derived total fat mass and four MRI-based measures (mean SAT and VAT across L1-L5, VAT/SAT ratio computed as means across L1-L5, and percent liver fat) were analyzed in relation to T2D status.

Using general linear regression, we estimated mean values and 95% confidence intervals (CI) of adiposity measures across the four categories of T2D status. Trends for the continuum from NG to T2D categories coded as 1–4 were computed to assess a dose-response relation for a gradient of metabolic disturbance. All models were adjusted for age at clinic visit, ethnic group, alcohol intake, physical activity, fasting hours, smoking status, and the HEI-2010 based on previous analyses in this study population.^13^^,^^19^ In addition, total DXA fat mass was included into models for VAT, the VAT/SAT ratio, and percent liver fat to adjust for overall adiposity. Spearman correlation coefficients indicated only weak associations of DXA total body with SAT, VAT, VAT/SAT ratio, and percent liver fat (*r*_s_ = 0.89, 0.46, −0.23, and 0.35; all *P* < 0.0001). Because of the known differences in body fat distribution and diabetes risk among racial/ethnic groups,^12^^,^^20^ stratified analyses by race/ethnicity were performed for the VAT/SAT ratio and percent liver fat. Finally, multinomial logistic regression using the same covariates as in the linear regression was applied to estimate prevalence odds ratios (POR) for 1 standard deviation (SD) of the VAT/SAT ratio in the total population and stratified by ethnic group. The POR describes the odds of belonging to the PT2D, UT2D, or T2D group as compared to NG for a 1 SD increase in the VAT/SAT ratio.

## RESULTS

Among 1,746 participants (51% women) with DXA and MRI imaging results (Table 1), the median age was 69.2 (interquartile range, 4.2) years, ranging from 69.2 to 77.4 years. Due to the recruitment strategy, 40% of participants were overweight and 30% obese. Overall, 315 (18%) were classified as T2D, 158 (9%) as UT2D, and 518 (30%) as PT2D while the remaining 755 (43%) were NG. These proportions differed significantly by ethnicity (*P* < 0.0001) with 31% Japanese Americans, 39% Native Hawaiians, 48% Whites, 46% Latinos, and 55% African Americans in the NG category. The prevalence of T2D and UT2D was lowest in whites (15%), followed by African Americans (26%) and Native Hawaiians (28%), and highest in Japanese Americans (32%) and Latinos (34%). Mean concentrations of FG, insulin, and HOMA-IR were lowest in the NG group, 20–30% higher in the PT2D group, and more than 50% higher among individuals with UT2D and T2D as compared to the NG group. In contrast, SAT and DXA total were similar across NG, PT2D, and UT2D categories and approximately 10% higher among T2D cases. The patterns for VAT and VAT/SAT were different again: low for NG, intermediate for PT2D and UT2D, and 40% higher in the T2D than the NG category. All differences in life-style factors and lab measures across the four categories were statistically significant except for total energy intake and smoking status.

**Table 1.** Characteristics of the study population at clinic visit^a^

Characteristic	Categories	All	NG	PT2	UT2D	T2D	*P*-value^b^
*N*		1,746	755 (43)	518 (30)	158 (9)	315 (18)	NA

Sex	Men	855	313 (37)	283 (33)	87 (10)	172 (20)	
	Women	891	442 (50)	235 (26)	71 (8)	143 (16)	<0.0001

Ethnicity	White	392	186 (48)	145 (37)	37 (9)	24 (6)	
	African American	287	159 (55)	91 (19)	16 (6)	56 (20)	
	Native Hawaiian	279	108 (39)	91 (33)	29 (10)	51 (18)	
	Japanese American	421	131 (31)	154 (37)	58 (14)	78 (18)	
	Latino	367	171 (46)	72 (20)	18 (5)	106 (29)	<0.0001

BMI status	<25 kg/m^2^	516	266 (51)	152 (30)	47 (9)	51 (10)	
	25–<30 kg/m^2^	705	299 (42)	217 (31)	63 (9)	126 (18)	
	≥30 kg/m^2^	525	190 (36)	149 (29)	48 (9)	138 (26)	<0.0001

Smoking status	Never	1,068	487 (46)	313 (29)	92 (9)	176 (16)	
	Past	678	268 (40)	205 (30)	66 (10)	139 (20)	0.05

Age	Years	69.2 (4.2)	69.2 (4.3)	69.9 (4.0)	68.5 (3.8)	69.7 (4.3)	0.0001

Alcohol intake	Drinks/day	0.07 (0.63)	0.09 (0.58)	0.10 (0.81)	0.07 (0.70)	0.03 (0.36)	0.03

Physical activity	Hours/day	1.18 (1.25)	1.18 (1.21)	1.18 (1.46)	1.18 (1.21)	0.93 (1.18)	0.02

Diet quality	HEI-2010 scores	73.4 (14.2)	74.4 (13.8)	72.9 (15.6)	71.5 (14.7)	71.0 (13.2)	<0.0001

Total energy intake	kcal/day	1,687 (1,003)	168 (1,007)	1,695 (953)	1,867 (971)	1,603 (1,012)	0.36

DXA total body fat	kg	24.3 (11.1)	24.0 (11.4)	23.1 (11.3)	23.6 (10.0)	26.7 (10.0)	<0.0001

VAT	cm^2^	158 (110)	133 (98)	160 (112)	169 (93)	200 (109)	<0.0001

SAT	cm^2^	212 (128)	213 (127)	205 (124)	207 (140)	216 (124)	<0.0001

VAT/SAT ratio		0.72 (0.67)	0.61 (0.55)	0.79 (0.73)	0.76 (0.65)	0.96 (0.81)	<0.0001

Liver fat	%	3.93 (4.30)	4.37 (2.68)	4.10 (4.41)	4.19 (5.18)	5.97 (6.26)	<0.0001

Fasting glucose	mg/dL	102 (29)	89 (13)	108 (12)	136 (14)	131 (52)	<0.0001

Fasting insulin	µU/mL	5.97 (5.41)	5.05 (4.38)	6.23 (5.44)	7.88 (6.47)	8.05 (6.49)	<0.0001

HOMA-IR		1.49 (1.61)	1.08 (0.99)	1.70 (1.49)	2.70 (2.35)	2.62 (2.42)	<0.0001

BMI, body mass index; DXA, dual X-ray absorptiometry; HEI-2010, Healthy Eating Index; HOMA-IR, Homeostatic Model Assessment of Insulin Resistance; NG, normoglycemic; PT2D, prediabetes (fasting glucose 100–125 mg/dL); SAT, subcutaneous adipose tissue; T2D, self-reported type 2 diabetes and/or taking diabetes medication; UT2D, undiagnosed T2D (fasting glucose >125 mg/dL); VAT, visceral adipose tissue.

^a^Medians (Interquartile Ranges) are shown except for sex, ethnicity, BMI, and smoking status where participant numbers (percent) are given.

^b^Obtained through chi-square tests or general linear models.

After adjustment for covariates, all adiposity measures except SAT showed significant positive trends across the NG to T2D continuum (Table 2). The adjusted mean values of BMI, DXA total body fat, and VAT were 10–20% higher in T2D than NG participants. Mean DXA total body fat levels were higher for those in the PT2D/UT2D than in the NG group (25.8 vs 24.9 kg) but highest in T2D cases (27.3 kg). The means of the VAT/SAT ratio were 0.81, 0.86, 0.89, and 1.00 (*P* < 0.0001) for the NG, PT2D, UT2D, and T2D groups, respectively. The largest difference of 40% was seen for percent liver fat with means of 4.93 (95% CI, 4.60–5.25) in the NG and 7.02 (95% CI, 6.53–7.51) in the T2D group (*P* < 0.0001).

**Table 2.** Adjusted means of adiposity measures by diabetes status

Adiposity measure^a^	Diabetes status	Mean	95% confidence interval		*P*_trend_^b^	*P*_interaction_^c^
BMI, kg/m^2^	NG	27.3	27.0	27.7		
	PT2D	28.2	27.8	28.6		
	UT2D	28.2	27.5	28.9		
	T2D	29.2	28.7	29.7	<0.0001	0.009

DXA total fat, kg	NG	24.9	24.4	25.5		
	PT2D	25.8	25.2	26.5		
	UT2D	25.8	24.7	26.9		
	T2D	27.3	26.5	28.1	<0.0001	0.03

SAT, cm^2^	NG	230	227	234		
	PT2D	232	228	236		
	UT2D	234	227	241		
	T2D	225	220	230	0.21	0.004

VAT, cm^2^	NG	157	153	162		
	PT2D	169	164	173		
	UT2D	167	158	175		
	T2D	190	184	196	<0.0001	0.09

VAT/SAT	NG	0.81	0.78	0.84		
	PT2D	0.86	0.83	0.90		
	UT2D	0.89	0.83	0.95		
	T2D	1.00	0.95	1.04	<0.0001	0.003

% liver fat	NG	4.93	4.60	5.25		
	PT2D	5.78	5.39	6.16		
	UT2D	5.67	5.00	6.34		
	T2D	7.02	6.53	7.51	<0.0001	0.11

BMI, body mass index; DXA, dual X-ray absorptiometry; NG, normoglycemic; PT2D, prediabetes (fasting glucose 100–125 mg/dL); SAT, subcutaneous adipose tissue; T2D, self-reported type 2 diabetes and/or taking diabetes medication; UT2D, undiagnosed T2D (fasting glucose >125 mg/dL); VAT, visceral adipose tissue.

^a^Obtained through general linear regression adjusted for age, sex, ethnicity, physical activity, alcohol intake, smoking status, HEI-2010, fasting hours, and DXA total body fat (except for BMI and total body fat models).

^b^*P*-value obtained from general linear model with diabetes status as a nominal ordinal variable (0–3).

^c^*P*-value obtained from interaction term between ethnicity and diabetes status (0–3).

**Figure 1.**
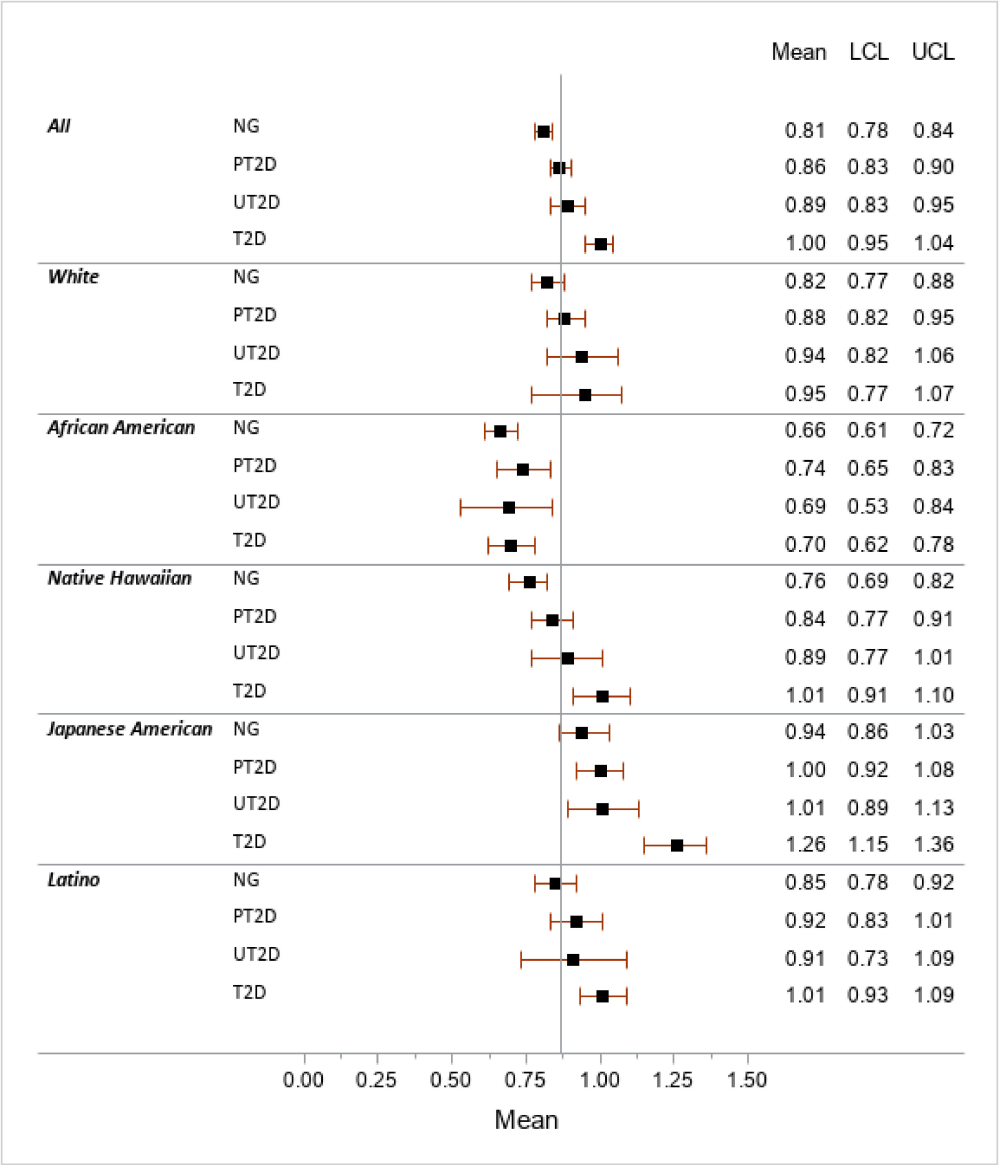
Ethnic-specific means of visceral to subcutaneous adipose tissue ratio by diabetes status^a^ LCL, lower confidence limit; NG, normoglycemic; POR, prevalence odds ratio; PT2D, prediabetes (fasting glucose 100–125 mg/dL); SAT, subcutaneous adipose tissue; T2D, self-reported type 2 diabetes and/or taking diabetes medication; UCL, upper confidence limit; UT2D, undiagnosed T2D (fasting glucose >125 mg/dL); VAT, visceral adipose tissue. ^a^Obtained through general linear regression adjusted for age, sex, physical activity, alcohol intake, smoking status, HEI-2010, DXA total body fat, and fasting hours stratified by ethnic group. The vertical line represents the overall mean of the VAT/SAT ratio (0.87).

Ethnic differences in VAT/SAT were visible across categories of glycemic status (Figure 1) with the highest ratios among Japanese Americans and the lowest among African Americans. The trend of the VAT/SAT ratio across glycemic categories was significant for all ethnic groups except African Americans. The highest VAT/SAT values (0.94, 1.00, 1.01, 1.26) were seen in Japanese Americans with a significant trend (*P* < 0.0001). Native Hawaiians showed lower means (0.76, 0.84, 0.89, 1.01) but also a significant trend (*P* < 0.0001). Latinos with T2D had a significantly higher VAT/SAT compared to the NG group (1.01 vs 0.85) with a significant trend across categories (*P* = 0.003) although the 95% CIs overlapped with PT2D and UT2D. The trends were weaker in whites (*P* = 0.04), whereas the low mean VAT/SAT values (range, 0.66–0.74) in African Americans did not differ by diabetes status (*P* = 0.67).

**Figure 2.**
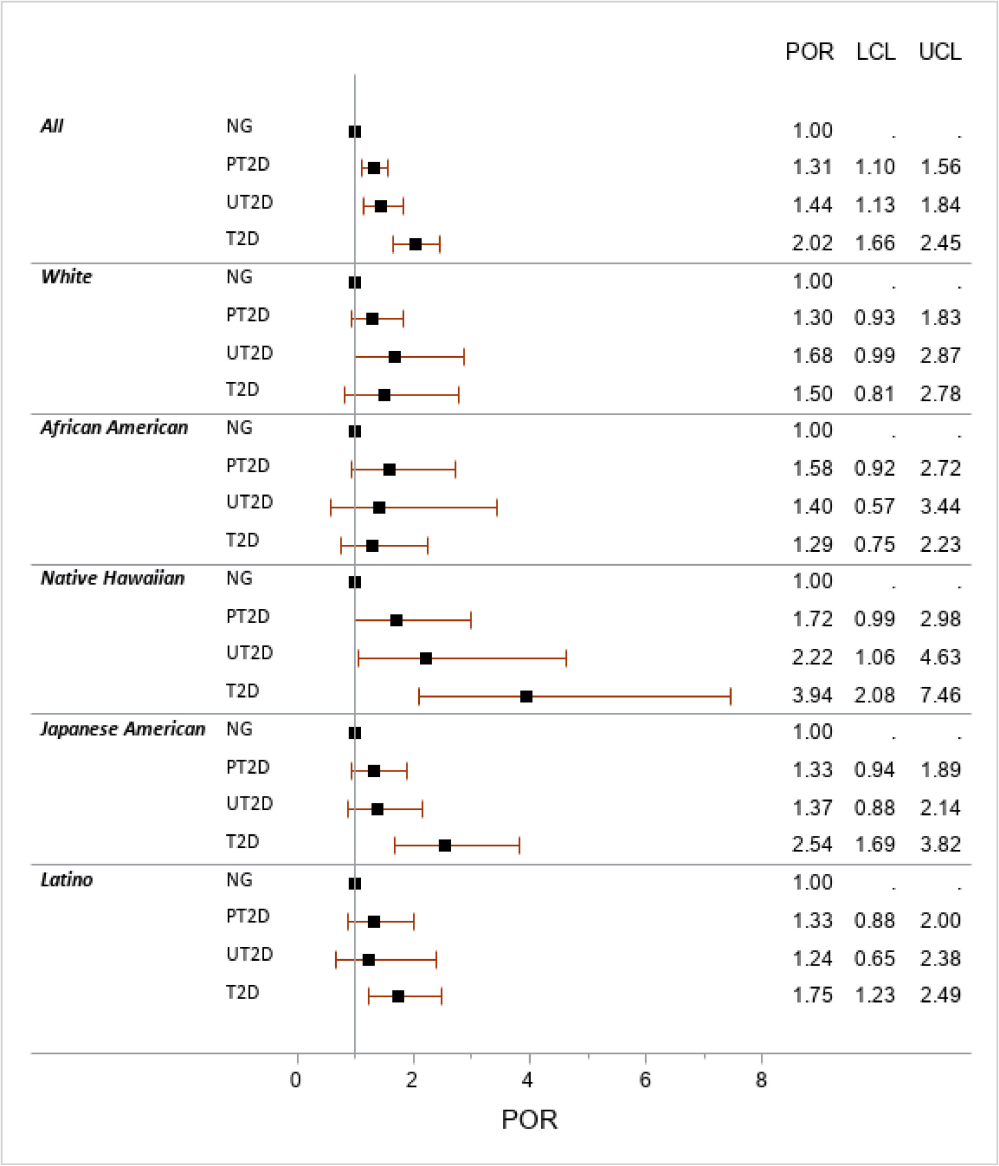
Ethnic-specific prevalence odds ratio of the VAT/SAT ratio by diabetes status^a^ LCL, lower confidence limit; NG, normoglycemic; POR, prevalence odds ratio; PT2D, prediabetes (fasting glucose 100–125 mg/dL); SAT, subcutaneous adipose tissue; T2D, self-reported type 2 diabetes and/or taking diabetes medication; UCL, upper confidence limit; UT2D, undiagnosed T2D (fasting glucose >125 mg/dL); VAT, visceral adipose tissue. ^a^Obtained through multinomial logistic regression for 1 standard deviation of the VAT/SAT ratio adjusted for age, sex, physical activity, alcohol intake, smoking status, Healthy Eating Index-2010, DXA total body fat, and fasting hours with NG as reference.

PORs for VAT/SAT (Figure 2) indicated that each additional SD of the VAT/SAT ratio doubled the odds of being in the T2D group for the entire study population. However, for African Americans, whites, and Latinos the respective risks were lower (29%, 50%, and 75%), while Native Hawaiians and Japanese Americans were 2.5 and 4 times more likely to report T2D per 1 SD of VAT/SAT. The PORs for the PT2D and UT2D groups were similar to each other and closer to NG than T2D.

**Figure 3.**
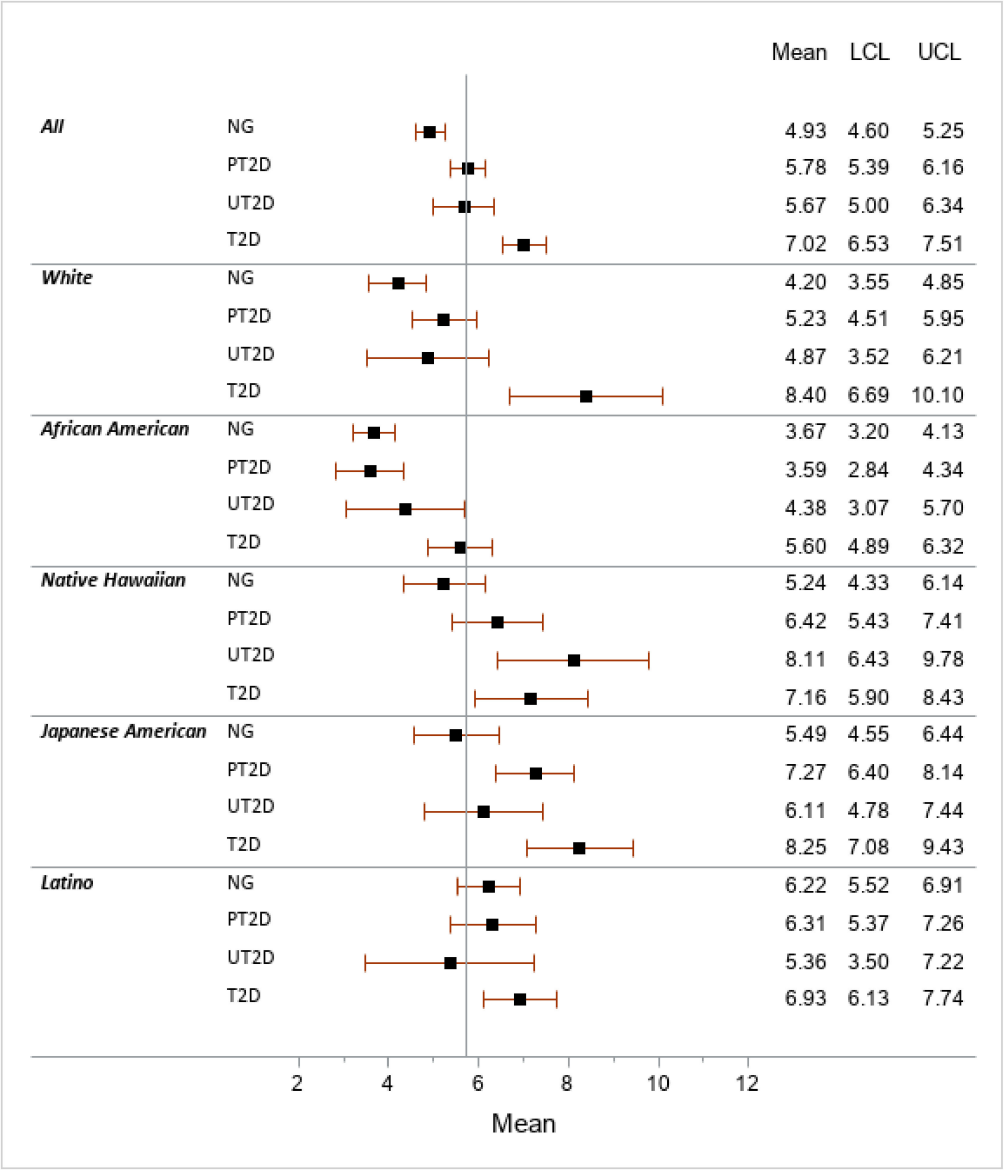
Ethnic-specific means of percent liver fat by diabetes status^a^ LCL, lower confidence limit; NG, normoglycemic; POR, prevalence odds ratio; PT2D, prediabetes (fasting glucose 100–125 mg/dL); SAT, subcutaneous adipose tissue; T2D, self-reported type 2 diabetes and/or taking diabetes medication; UCL, upper confidence limit; UT2D, undiagnosed T2D (fasting glucose >125 mg/dL); VAT, visceral adipose tissue. ^a^Obtained through general linear regression adjusted for age, sex, physical activity, alcohol intake, smoking status, HEI-2010, DXA total body fat, and fasting hours stratified by ethnic group. The vertical line represents the overall mean of the percent liver fat (5.71).

Significantly higher values of percent liver fat across T2D categories were seen for all ethnic groups except Latinos (Figure 3), with *P*-values for trend of <0.0001, <0.0001, 0.005, 0.001, and 0.32 for whites, African Americans, Native Hawaiians, Japanese Americans, and Latinos, respectively. The mean values were greatest among Japanese Americans and Native Hawaiians, but the largest difference across categories was seen in whites with more than twice as much liver fat in the T2D than the NG group.

## DISCUSSION

The current analysis in older adults observed a significant trend of higher VAT, VAT/SAT, and percent liver fat across the continuum of glycemic status (NG, PT2D, UT2D, to T2D) after adjustment for DXA-derived total body fat indicating that the presence of ectopic fat is associated with T2D etiology beyond the influence of obesity. This association differed by ethnic group. The VAT/SAT ratio showed the greatest difference across the four categories for Japanese Americans (0.32) and Native Hawaiians (0.25), whereas the low values for African Americans did not vary by glycemic status. For each SD of VAT/SAT, Native Hawaiians were 4 times as likely and Japanese Americans 2.5 times as likely to report T2D. Percent liver fat increased from NG to T2D for all ethnic groups except Latinos. The findings among Japanese Americans highlight that they, like other Asians, have high levels of the VAT and liver fat despite their low BMIs.^21^ Therefore, it appears that T2D development T2D is driven more by the presence of ectopic fat than just obesity as in other groups. The similar results among Native Hawaiians can be contributed to their large proportion of Asian admixture.^22^

Many reports are in general agreement with the PORs in the current study. The Netherlands Epidemiology of Obesity Study showed ORs of 1.72 and 2.33 for men and women per 1 SD VAT.^23^ In an international study from 29 countries,^24^ higher VAT and liver fat were found with disturbed glucose metabolism; a 1 SD increase in VAT showed an OR of 1.80 (95% CI, 1.35–2.42) in men and 1.73 (95% CI, 1.25–2.41) in women, Similarly, a meta-analysis of three cross-sectional investigations in whites and Koreans reported 2- and 4-fold increases in T2D for every additional 1 kg of VAT measured by DXA among men and women.^4^ Prospective studies assessing VAT through imaging are rare, but in a pooled analysis of 14 prospective studies, the waist-to-hip ratio adjusted for BMI as a marker for central adiposity was a strong predictor of T2D (OR 1.82; 95% CI, 1.38–2.42) per 1 SD.^25^ In the Dallas Heart study,^26^ MRI-derived VAT was significantly associated with the prevalence of diabetes (OR 1.41; 95% CI, 1.06–1.87), but SAT was not (OR 1.02; 95% CI, 0.67–1.55). The associations were similar across whites, African Americans, and Hispanics. One of the few prospective studies examining image-derived VAT and the risk of T2D was conducted among Japanese Americans in Seattle.^27^ Intra-abdominal fat area (IAF) as assessed by CT scan increased by a mean of 11 cm^2^ during follow-up of 436 Japanese Americans without T2D; both baseline IAF and an increase of 1 SD in IAF were associated with a more than 1.6-fold increase in T2D after adjustment for covariates. These values are not directly comparable to the POR of 2.5 among the Japanese Americans in the current study as different VAT measures were applied.

The higher prevalence of T2D among non-whites versus whites has been documented extensively in the MEC^9^ and other populations around the world.^10^^,^^11^ Whereas T2D incidence in persons with Japanese and other Asian ancestry is high despite relatively low BMI levels, the T2D burden among Native Hawaiians and African Americans is largely attributable to the high prevalence of obesity.^9^ Although the VAT/SAT ratio in African Americans is low, obesity rates are high in the MEC^14^ and national samples^28^ explaining the high T2D incidence. The high prevalence of UT2D observed in Japanese Americans is difficult to evaluate. It may be due the relatively low BMI,^12^ which does not prompt as much testing as in ethnic groups with high obesity rates although health care access and utilization in Hawaii are high. The fact that the UT2D group is closer to the PT2D than the T2D group in terms of ectopic fat suggests that the elevated glucose levels may also just be a chance finding. Among Native Hawaiians, health seeking behavior or access to preventive care services may be responsible for the higher UT2D rates.^29^

Ethnic differences for the association of body fat deposition and incident T2D were shown in a British cohort; VAT estimated from anthropometry-based prediction equations was higher in South Asians and lower in African Caribbeans than Europeans.^30^ As to biologic mechanisms, a UK Biobank Study suggested that a genetically determined adiposity phenotype might be related to lower ectopic liver fat and lower risk for T2D.^31^ Certain alleles (*PPARG*, *GRB14*, and *IRS1*) were associated with higher adiposity but presented with a favorable metabolic profile such as a lower VAT/SAT ratio and a lower risk of T2D. This pattern is consistent with the idea that storing excess triglycerides in metabolically low-risk depots is beneficial^31^^–^^33^ and agree with higher rates of NAFLD in T2D patients and an elevated risk of developing T2D among persons with NAFLD.^2^^,^^34^ T2D and NAFLD may, however, share insulin resistance as a common pathophysiological mechanism, and each of the two conditions may affect the development of the other, but it has not been determined whether one disease is the cause of the other or if they have a shared cause in lifestyle factors.^35^

Strengths of the current study include the multiethnic population with Native Hawaiians and Japanese Americans known to have high rates of T2D,^12^ the exclusion of participants with type 1 diabetes, and the quantification of body fat measures through DXA and MRI imaging. However, a number of limitations also need to be considered. A single measure of FG without a glucose tolerance test or an HbA1c measure is not optimal to assess glycemic status.^36^ Most likely, impaired glucose tolerance was not detected in some of our participants or some of the elevated FG levels were random events and should not have been classified as PT2D or UT2D; both scenarios would result in misclassification. The challenge of classifying T2D status may be aggravated among African Americans because fasting hyperglycemia appears to be a less common early sign of T2D as gluconeogenesis remains low.^37^ Due to the cross-sectional design, causality cannot be answered; we do not know if VAT was responsible for T2D development or if a common factor or genetic susceptibility was responsible. DXA and MRI scans taken before the diagnosis of T2D would have provided more insight into the role of body fat measures during T2D development, but as of now, few prospective cohorts have assessed imaging-based VAT in large populations.^3^ Given the ethnic and age distribution of this study, the current results may not be generalizable to other populations with different ancestries and younger age groups. The strict inclusion criteria for the APS and the low participation rate further limit generalizability.

The current analysis adds to evidence that body fat distribution plays a role in T2D status beyond the elevated risk due to obesity. The notable differences across ethnic groups represent novel findings, in particular for T2D etiology among Japanese Americans and Native Hawaiians with Asian admixture.^38^ As shown in different populations, there is evidence that individuals of Japanese and other Asian ancestry are more likely to have dysfunctional or insufficient subcutaneous depots and a higher propensity to accumulate ectopic fat.^32^^,^^33^^,^^39^ This knowledge is important for early detection and prevention among these populations who experience some of the highest T2D incidence in the world.
